# Spin-Orbital Conversion with the Tight Focus of an Axial Superposition of a High-Order Cylindrical Vector Beam and a Beam with Linear Polarization

**DOI:** 10.3390/mi13071112

**Published:** 2022-07-15

**Authors:** Victor Kotlyar, Sergey Stafeev, Vladislav Zaitsev, Elena Kozlova

**Affiliations:** 1 Image Processing Systems Institute of the RAS—Branch of FSRC “Crystallography & Photonics” of the RAS, 151 Molodogvardeyskaya St., 443001 Samara, Russia; kotlyar@ipsiras.ru (V.K.); sergey.stafeev@gmail.com (S.S.); zaicev-vlad@yandex.ru (V.Z.); 2 Technical Cybernetics Department, Samara National Research University, Moskovskoye Shosse 34, 443086 Samara, Russia

**Keywords:** tight focusing, Richards–Wolf formulas, Stokes vector, spin angular momentum, vortex energy flow, Poynting vector

## Abstract

In this paper, spin-orbital conversion in the tight focus of an axial superposition of a high-order (order *m*) cylindrical vector beam and a beam with linear polarization is theoretically and numerically considered. Although such a beam does not have a spin angular momentum in the initial plane and the third projection of its Stokes vector is equal to zero, subwavelength local regions with a transverse vortex energy flow and with the non-zero third Stokes projection (the longitudinal component of the spin angular momentum) are formed in the focal plane for an odd number *m*. This means that such a beam with an odd *m* has regions of elliptical or circular polarization with alternating directions of rotation (clockwise and counterclockwise) in the focus. For an even *m*, the field is linearly polarized at every point of the focal plane, and the transverse energy flux is absent. These beams can be used to create a micromachine in which two microparticles in the form of gears are captured in the focus of the beam into neighboring local areas in which the energy flow rotates in different directions, and therefore, these gears will also rotate in different directions.

## 1. Introduction

Cylindrical vector fields, including those of higher orders, are well known [1,2]. They are an example of inhomogeneously polarized light beams in the cross-section of which the local linear polarization vector changes its direction from point to point. Cylindrical beams of low orders are called beams with radial and azimuthal polarization [1]. Such beams can, for example, be formed using two half-wave plates rotated relative to each other [1], multisection polarizers [3], metasurfaces [4], quarter-wave plates and a light modulator [5]. Cylindrical vector beams (CVB) are used in particle micromanipulation [6], microscopy [7] and quantum informatics [8,9].

CVBs of any order do not have a spin angular momentum (SAM), and the third component of the Stokes vector is zero. This means that both in the initial plane and in any other section of the beam during its propagation the polarization is locally linear. It has recently been shown that local subwavelength regions with circular and elliptical polarization appear in the focus of CVBs of a fractional-order [10]. The optical effect of spin-orbital conversion is known in laser beams when a transverse vortex energy flux is formed during the tight focusing of an ordinary Gaussian beam with circular polarization. That is, such a beam has an orbital angular momentum (OAM) in the focus [11]. However, the occurrence of a local SAM in the focus of fractional-order CVBs [10] does not have an explanation since such a beam does not possess a SAM in the initial plane. Another disadvantage of the research [10] is that it is impossible to apply the analytical theory of Richards–Wolf [12] to describe the electromagnetic field in a sharp focus due to the fractional-order of the beam. In [13], a modification of a cylindrical vector field formed by a superposition of such a beam with a beam of uniform linear polarization was considered. However, in [13], the tight focusing of such a superposition was not considered.

In this paper, all six projections of electric and magnetic field vectors in a tight focus of the superposition of a cylindrical vector field and a uniform field with linear polarization are calculated theoretically and numerically using the Richards–Wolf approach [12]. Energy fluxes (projections of the Poynting vector), intensity distributions and Stokes components are also obtained. It is shown that local transverse vortex energy fluxes and subwavelength regions with an elliptical and circular polarization are formed in the focus of such a field with an odd integer order.

The motivation for writing this paper is that we considered a vector light field without spin, and therefore, the effect of spin-orbital conversion is not applicable to this field, and the total SAM and OAM are equal to zero in the focus of such a field. However, since the symmetry of the *m*th order initial vector field is broken due to the interference with a linearly polarized light field, local regions with a vortex transverse energy flow and a circular polarization appear in the focus. Moreover, transverse energy flow and circular polarization vector rotation directions are mutually opposite in neighboring regions. Such a light field can be used to create a micromachine in which two microparticles in the form of gears are captured in the focus of the beam into neighboring local areas in which the energy flow rotates in different directions, and therefore, these gears will also rotate in different directions. In addition, the considered light field can be fully analyzed theoretically, and the number and the size of local areas in the focus, in which the vortex motion of energy occurs, can be predicted. It should be noted that a cylindrical vector field of the *m*th order by itself (without superposition with a linearly polarized field) does not exhibit such properties and does not form local transverse energy vortices with the opposite direction of rotation in the focus.

## 2. Theoretical Investigation

### 2.1. Projections of Electric and Magnetic Fields Vectors in the Focal Plane

We consider an initial light field with a non-uniform polarization, the Jones vector for the electric and magnetic fields of which has the form:

(1) $$\begin{array}{l} E_{m}\left(\varphi ,a\right)=\left(\begin{array}{l} \cos\left(m\varphi \right)-a\\ \sin\left(m\varphi \right) \end{array}\right)=\left(\begin{array}{l} \cos\left(m\varphi \right)\\ \sin\left(m\varphi \right) \end{array}\right)-a\left(\begin{array}{l} 1\\ 0 \end{array}\right),\\ H_{m}\left(\varphi ,a\right)=\left(\begin{array}{l} -\sin\left(m\varphi \right)\\ \cos\left(m\varphi \right)-a \end{array}\right), \end{array}$$

where (*r*, φ) are polar coordinates in the initial plane and *a* is a real number. This light field was considered in [13]. It was shown that the field (1) has the Poincaré–Hopf polarization singularity index η [14], which is η = *m* for $\left|a\right|<1$, η = *m*/2 for $\left|a\right|=1$ and η = 0 for $\left|a\right|>1$. The light field (1) is an axial superposition of two well-known light fields: a cylindrical vector field of the *m*th order and a light field with the linear polarization directed along the horizontal axis. The real number *a* determines the polarization singularity index of the field (1) and the distribution of intensity, the energy flux and the density of SAM in the tight focus of the field (1).

For *a* = 0, the field (1) is a well-known CVB of a high order [2,15]. The beam (1) at *a* = 0 has an inhomogeneous polarization, and the polarization is locally linear at each point of the beam cross-section. The polarization of *m*th order CVB is also linear in each point of the focus. The purpose of this study is to show the presence of local areas in the focus of the field (1) with *a* ≠ 0, where a transverse energy flow (the energy circulates in a closed loop) is formed, and the longitudinal projection of the SAM vector is different from zero, i.e., there is an elliptical and a circular polarization.

Using the Richards–Wolf formalism [12], explicit expressions for all projections of electric and magnetic field vectors in the tight focus of the light field (1) were obtained:

(2) $$\begin{array}{l} E_{x}\left(r,\varphi \right)=i^{m-1}\left(\cos\left(m\varphi \right)I_{0,m}+\cos\left(\left(m-2\right)\varphi \right)I_{2,m-2}\right)+ia\left(I_{0,0}+\cos\left(2\varphi \right)I_{2,2}\right),\\ E_{y}\left(r,\varphi \right)=i^{m-1}\left(\sin\left(m\varphi \right)I_{0,m}-\sin\left(\left(m-2\right)\varphi \right)I_{2,m-2}\right)+ia\sin\left(2\varphi \right)I_{2,2},\\ E_{z}\left(r,\varphi \right)=2i^{m}\cos((m-1)\varphi )I_{1,m-1}+2a\cos\varphi I_{1,1},\\ H_{x}\left(r,\varphi \right)=-i^{m-1}\left(\sin\left(m\varphi \right)I_{0,m}+\sin\left(\left(m-2\right)\varphi \right)I_{2,m-2}\right)+ia\sin\left(2\varphi \right)I_{2,2},\\ H_{y}\left(r,\varphi \right)=-i^{m-1}\left(-\cos\left(m\varphi \right)I_{0,m}+\cos\left(\left(m-2\right)\varphi \right)I_{2,m-2}\right)+ia\left(I_{0,0}-\cos\left(2\varphi \right)I_{2,2}\right),\\ H_{z}\left(r,\varphi \right)=-2i^{m}\sin\left(\left(m-1\right)\varphi \right)I_{1,m-1}+2ia\sin\varphi I_{1,1}. \end{array}$$

In (2), functions *I*_ν,µ_ depend only on the radial variable *r* and are equal to the expression:

(3) $$I_{\nu ,\mu }=\left(\frac{\pi f}{\lambda }\right)\int_{0}^{\theta_{0}}\sin^{\nu +1}\left(\frac{\theta }{2}\right)\cos^{3-\nu }\left(\frac{\theta }{2}\right)\cos^{1/2}\left(\theta \right)A\left(\theta \right)e^{ikz\cos\theta }J_{\mu }\left(x\right)d\theta ,$$

where *k* is the wave number of light, λ is the wavelength of light, f is the focal length of an ideal spherical lens that forms the focus, z is an optical axis (*z* = 0 is the focal plane), *x* = *kr*sin θ, *J*_μ_(*x*) is the Bessel function of the first kind and of the μth order, *NA* = sin θ_0_ is the numerical aperture of an aplanatic optical system and *A*(θ) is any real function that describes the input field amplitude, which has an axial symmetry (plane wave, Gaussian beam, Bessel-Gaussian beam). For the integrals *I*_ν,µ_ (3), the first index ν = 0, 1, 2 describes the type of the integral, and the second index μ = 0, 1, 2, …, *m* is equal to the order of the Bessel function.

Each projection of electric and magnetic field vectors (2) is the sum of two beams vectors projections: a cylindrical vector field of the *m*th order and a light field with linear polarization. This is easy to verify if we recall what projections an electromagnetic field with linear polarization directed along the horizontal axis has in the focus [12]:

(4) $$\begin{array}{l} E_{Lx}\left(r,\varphi \right)=-i\left(I_{0,0}+\cos\left(2\varphi \right)I_{2,2}\right),\\ E_{Ly}\left(r,\varphi \right)=-i\sin\left(2\varphi \right)I_{2,2},\\ E_{Lz}\left(r,\varphi \right)=-2\cos\varphi I_{1,1},\\ H_{Lx}\left(r,\varphi \right)=-i\sin\left(2\varphi \right)I_{2,2},\\ H_{Ly}\left(r,\varphi \right)=-i\left(I_{0,0}-\cos\left(2\varphi \right)I_{2,2}\right),\\ H_{Lz}\left(r,\varphi \right)=-2a\sin\varphi I_{1,1}. \end{array}$$

### 2.2. The Intensity Distribution in the Focal Plane

Based on the obtained amplitudes of the electric field vector projections in the focus (2), it is possible to derivate expressions for the intensity and its components along the Cartesian axes. It should be noted that the expressions for the intensity with even and odd numbers *m* will be different. Indeed, formula (2) implies the expressions for $I=I_{x}+I_{y}+I_{z}={\left|E_{x}\right|}^{2}+{\left|E_{y}\right|}^{2}+{\left|E_{z}\right|}^{2}$:

(5) $$I_{x}=\left\{\begin{array}{l} {\left[a{\left(-1\right)}^{p+1}\left(I_{0,0}+\cos\left(2\varphi \right)I_{2,2}\right)+\cos\left(m\varphi \right)I_{0,m}+\cos\left(\left(m-2\right)\varphi \right)I_{2,m-2}\right]}^{2},\;m=2p,\\ {\left[a\left(I_{0,0}+\cos\left(2\varphi \right)I_{2,2}\right)\right]}^{2}+{\left[\cos\left(m\varphi \right)I_{0,m}+\cos\left(\left(m-2\right)\varphi \right)I_{2,m-2}\right]}^{2},\;m=2p+1, \end{array}\right.$$

(6) $$I_{y}=\left\{\begin{array}{l} {\left[a{\left(-1\right)}^{p+1}\sin\left(2\varphi \right)I_{2,2}+\sin\left(m\varphi \right)I_{0,m}-\sin\left(\left(m-2\right)\varphi \right)I_{2,m-2}\right]}^{2},\;m=2p,\\ {\left[a\sin\left(2\varphi \right)I_{2,2}\right]}^{2}+{\left[\sin\left(m\varphi \right)I_{0,m}-\sin\left(\left(m-2\right)\varphi \right)I_{2,m-2}\right]}^{2},\;m=2p+1, \end{array}\right.$$

(7) $$I_{z}=\left\{\begin{array}{l} 4{\left[a{\left(-1\right)}^{p}\cos\left(\varphi \right)I_{1,1}+\cos\left(\left(m-1\right)\varphi \right)I_{1,m-1}\right]}^{2},\;m=2p,\\ 4{\left[a\cos\left(\varphi \right)I_{1,1}\right]}^{2}+4{\left[\cos\left(\left(m-1\right)\varphi \right)I_{1,m-1}\right]}^{2},\;m=2p+1. \end{array}\right.$$

It can be seen from (7) that for *m* = 2 and *p* = 1, the intensity is equal to the simple expression:

(8) $$I_{2z}\left(r,\varphi \right)=4\cos^{2}\left(\varphi \right)I_{1,1}^{2}{\left(1-a\right)}^{2}.$$

It can be seen from (8) that the longitudinal intensity (8) is zero at *a* = 1, and it has two local maxima on the horizontal *x-*axis (φ = 0 and φ = *π*) at *a* ≠ 1.

Next, we obtained the expressions for the total intensity in the focus at *m* = 2*p* + 1 because, as it will be shown later, the transverse energy fluxes and the longitudinal projection of the SAM arise in the focus only for odd numbers *m*:

(9) $$\begin{array}{l} I=a^{2}\left[I_{0,0}^{2}+I_{2,2}^{2}+2I_{1,1}^{2}+2\cos2\varphi \left(I_{0,0}I_{2,2}+I_{1,1}^{2}\right)\right]+\\ +\left[I_{0,m}^{2}+I_{2,m-2}^{2}+2I_{1,m-1}^{2}+2\cos\left(2\left(m-1\right)\varphi \right)\left(I_{0,m}I_{2,m-2}+I_{1,m-1}^{2}\right)\right]. \end{array}$$

The expressions for the intensity (5) and (9) for an arbitrary *m* contains a term $a^{2}I_{0,0}^{2}$ > 0, in which, according to the integral (3), the zero-order Bessel function is used as one of the factors. Therefore, the intensity will be different from zero (there will be a local maximum) on the optical axis (at *r* = 0) since *J*_0_(0) = 1. The arguments of the cosines are even in the expression (9) for the intensity. This means that the intensity pattern, although it does not have a radial symmetry, has an axial symmetry, i.e., I(r,φ)=I(r,φ+π). Additionally, it can be seen from (5) that the intensity *I_x_* will have a maximum on the optical axis due to the term $a^{2}I_{0,0}^{2}$, and it follows from (6) that *I_y_* will have a zero on the optical axis. It should also be noted that the intensity pattern *I_y_* will have 2*m* local maxima since the expression for *I_y_* contains the squared cos(*m*φ). The total intensity (9) will have 2(*m* − 1) local maxima (except the intensity maximum on the optical axis) since formula (9) has cos(2(*m* − 1)φ). These conclusions will be confirmed by modeling.

### 2.3. The Energy Flux Density in the Focal Plane

In this section, the expressions for three projections of the Poynting vector in the focus of the light field (1) are obtained. It is known [15,16] that a cylindrical vector field of any order with *a* = 0 does not have a spin or vortex energy flows both in the initial plane (*E_x_*, *E_y_*) = (cos(*m*φ), sin(*m*φ)) and in the focus (2). The longitudinal projections of the SAM and the OAM vectors in the focus are zero at each point. Below, we show that the superposition of a CVB and a light field with linear polarization (2) has a local spin and a vortex energy flux. The Poynting vector is provided by the following formula [12]:

(10) P=c2πReE*×H,

where **E** and **H** are vectors of electric and magnetic fields, * is a complex conjugation, × is a vector multiplication and *c* is the light speed in a vacuum. Further, the constant *c*/(2π) will be ignored. We substituted the expressions for the projections of the electromagnetic field in the focus (2) into expression (10) and obtained:

(11) $$P_{x}\left(r,\varphi \right)=\left\{\begin{array}{l} 2a{\left(-1\right)}^{\left(m-1\right)/2}\left[\cos\left(\left(m-1\right)\varphi \right)\left(I_{1,1}I_{2,m-2}-I_{0,0}I_{1,m-1}\right)+\right.\\ \left.\cos\left(\left(m+1\right)\varphi \right)\left(I_{2,2}I_{1,m-1}-I_{1,1}I_{0,m}\right)\right],\;m=2p+1,\\ 0,\;m=2p,\;p=0,1,2,\ldots \end{array}\right.$$

(12) $$P_{y}\left(r,\varphi \right)=\left\{\begin{array}{l} 2a{\left(-1\right)}^{\left(m-1\right)/2}\left[-\sin\left(\left(m-1\right)\varphi \right)\left(I_{1,1}I_{2,m-2}-I_{0,0}I_{1,m-1}\right)+\right.\\ \left.\sin\left(\left(m+1\right)\varphi \right)\left(I_{2,2}I_{1,m-1}-I_{1,1}I_{0,m}\right)\right],\;m=2p+1,\\ 0,\;m=2p,\;p=0,1,2,\ldots \end{array}\right.$$

(13) $$P_{z}\left(r,\varphi \right)=\left\{\begin{array}{l} a^{2}\left(I_{0,0}^{2}-I_{2,2}^{2}\right)+\left(I_{0,m}^{2}-I_{2,m-2}^{2}\right)+\\ 2a{\left(-1\right)}^{p}\cos\left(m\varphi \right)\left(I_{2,2}I_{2,m-2}-I_{0,0}I_{0,m}\right),\;m=2p,\\ a^{2}\left(I_{0,0}^{2}-I_{2,2}^{2}\right)+\left(I_{0,m}^{2}-I_{2,m-2}^{2}\right),\;m=2p+1,\;p=0,1,2,\ldots \end{array}\right.$$

It can be seen from (13) that the distribution of the Poynting vector longitudinal component for odd numbers *m* has a circular symmetry in the focus. For an even number *m*, it depends on the polar angle and has *m* maxima when traveling around the optical axis. It can be seen from (11) and (12) that the transverse energy flow takes place only for odd numbers *m* and is equal to zero for an even *m*. To characterize the transverse energy flux in the focus in more detail, we proceed to the polar projections of the transverse energy flux vector. Using the transition from the Cartesian projections of the Poynting vector to polar:

(14) $$\begin{array}{l} P_{r}=P_{x}\cos\varphi +P_{y}\sin\varphi ,\\ P_{\varphi }=-P_{x}\sin\varphi +P_{y}\cos\varphi , \end{array}$$

from (11) and (12), we find the transverse components of the Poynting vector in the tight focus of the field (1) in polar coordinates for odd numbers *m* (for even numbers *m*, the Poynting vector components are equal to zero):

(15) $$\begin{array}{l} P_{r}=2a{\left(-1\right)}^{p}\cos\left(m\varphi \right)Q_{1}\left(r\right),\;m=2p+1,\\ Q_{1}(r)=I_{1,m-1}\left(I_{2,2}+I_{0,0}\right)-I_{1,1}\left(I_{2,m-2}+I_{0,m}\right),\\ P_{\varphi }=2a{\left(-1\right)}^{p}\sin\left(m\varphi \right)Q_{2}\left(r\right),\;m=2p+1,\\ Q_{2}(r)=I_{1,m-1}\left(I_{2,2}-I_{0,0}\right)+I_{1,1}\left(I_{0,m}-I_{2,m-2}\right). \end{array}$$

It can be seen from (15) that the transverse energy flow rotates non-uniformly at different radii, and for different *p*, the rotation occurs counterclockwise or clockwise. The irregularity lies in the fact that the transverse vector of the energy flux rotates around the optical axis not tangentially to some circle but at a different angle to some circle. There are 2*m* subwavelength regions on a circle centered on the optical axis in which the transverse energy flow rotates along a closed trajectory. It follows from Equations (11) and (12) that the transverse flow changes sign 2(*m* + 1) times per complete rotation due to the presence of terms with sin(*m* + 1) or with cos(*m* + 1)φ, which change sign 2(*m* + 1)φ times, in these Equations. Additionally, it follows from (15) that 2*m* local areas in which the energy will rotate since cos(*m*φ) and sin(*m*φ) factors are in (15) will be formed in the focus. Moreover, the energy flow rotates in different directions (clockwise or counterclockwise) in neighboring areas. The integration of radial and azimuthal energy fluxes in (15) over the angle φ gives zero. This means that the total transverse energy flux is zero in the focus.

### 2.4. The Density of the Stokes Vector in the Focal Plane

In this section, we find the projections of the Stokes vector in the focus of the initial vector field (1). The components of the Stokes vector **S** are calculated by the formulas [17]:

(16) S1=Ex2−Ey2Ex2+Ey2,S2=2ReEx*EyEx2+Ey2,S3=2ImEx*EyEx2+Ey2,s1=Ex2−Ey2,s2=2ReEx*Ey,s3=2ImEx*Ey,

where Re and Im determine the real and the imaginary parts of a complex number. In (16), the small letters (*s*_1_, *s*_2_, *s*_3_) denote the unnormalized components of the Stokes vector. The normalized Stokes vector, as it can be seen from (16), has a unit length $S_{1}^{2}+S_{2}^{2}+S_{3}^{2}=1$. Due to the cumbersomeness of the expressions, and in order to find out whether the circular polarization will be in focus, we obtained expressions only for the third Stokes projection without normalization, i.e., we calculated a function in the form s3=2ImEx*Ey. It should be preliminarily noted that the third component of the Stokes vector is proportional to the longitudinal projection of the SAM [16]:

(17) S=116πωImE*×E,

where ω is a cyclic frequency of light. Further, the constant 1/(16πω) will be ignored. It can be seen from (17) that the longitudinal component of the SAM (without taking into account the constant) coincides with the unnormalized third component of the Stokes vector:

(18) s3=Sz=2ImEx*Ey.

Substituting the projections of the electric field (2) into (18), we obtain:

(19) $$s_{3}=S_{z}\left(r,\varphi \right)=\left\{\begin{array}{l} 2a{\left(-1\right)}^{\left(m-1\right)/2}\left[\sin\left(\left(m-2\right)\varphi \right)\left(I_{0,0}I_{2,m-2}-I_{2,2}I_{0,m}\right)-\right.\\ \left.\sin\left(m\varphi \right)\left(I_{0,0}I_{0,m}-I_{2,2}I_{2,m-2}\right)\right],\;m=2p+1,\\ 0,\;m=2p,\;p=0,1,2,\ldots \end{array}\right.$$

It can be seen from (19) that there are no regions with a circular (elliptical) polarization in the focus of the field (1) for an even *m*. If *m* is odd and *a* ≠ 0, then there are 2*m* local regions in the focus, in which the light has an elliptical polarization. It should be noted from (15) that for an odd *m*, there are also 2*m* local regions of transverse vortex energy flows in the focus. A comparison of (15) and (19) shows that the number of regions with a transverse vortex energy flow in the focus is 2*m* and is equal to the number of regions with an elliptical polarization. Moreover, the direction of the transverse energy flow rotation is different in neighboring regions, just as the direction of the polarization vector rotation alternates in neighboring regions. Since the field (1) does not have the transverse energy flow and the longitudinal SAM in the initial plane, both the total (over the entire focal plane) longitudinal SAM and the total transverse energy flow must be equal to zero in the focus. It should be noted that if we integrate the spin density (19), i.e., the longitudinal component of the SAM, over the entire beam cross-section in the focus, then the integrals over the angle φ will give zero, and the total beam spin (1) in the focus, as in the initial plane, will be equal to zero:

$$ss_{3}=\int_{0}^{\infty }\int_{0}^{2\pi }s_{3}\left(r,\varphi \right)rdrd\varphi =0.$$

To compare the theory and simulation results, we derive an expression for *s*_2_ only for the even number *m* = 2*p*, *p* = 0, 1, 2, …:

(20) $$\begin{array}{l} s_{2}=\sin\left(2\varphi \right)\left[2I_{0,m}I_{2,m-2}+a^{2}I_{2,2}\left(I_{0,0}+\cos\left(2\varphi \right)I_{2,2}\right)\right]\\ \;\sin\left(2m\varphi \right)I_{0,m}^{2}-\sin\left(2\left(m-2\right)\varphi \right)I_{2,m-2}^{2},\;m=2p. \end{array}$$

It can be seen from (20) that the distribution of *s*_2_ in the focus will be axisymmetric since all arguments of the cosines and sines are even. The maximum argument in (20) has sin(2*m*φ) which is equal to 2*m*. Therefore, the number of sign changes for the function *s*_2_ will be equal to 4*m*.

The mechanism of an even number of local energy fluxes vortices formation in the focused field (1) with a CVB of an odd order can be described as follows. First, the polarization singularity index of the field (1) becomes half-integer *η* = *m*/2 only when *m* is odd and *a* = 1. Second, there are no transverse energy fluxes in the focus (11), (12) for even *m*. The half-integer polarization singularity index leads to the situation when the initial light field has *m* lines of the polarization singularity emanating from the center (the direction of the linear polarization is not determined on the singularity lines) and dividing the beam cross-section into *m* parts. In each of these parts (between two adjacent singularity lines), two local regions are formed in the focus (there are 2*m* such regions in the focus), in which the polarization is circular (elliptical) with different signs (left and right). This also follows from the expression (19). On the other hand, the presence of regions with non-zero spin density (17) in the focus, according to the effect of spin-orbital interaction, should simultaneously generate local regions with a vortex transverse energy flow (the non-zero longitudinal projection of the OAM). In total, the number of such regions with a vortex energy flow should be equal to the number of regions with the circular polarization, that is, equal to 2*m*. The number of local regions with a vortex transverse energy flow must be even to give the zero total OAM, and the energy flow rotation direction in neighboring areas should be opposite.

The presence of local regions with a vortex energy flow in the focus can be explained in another way. The field (1) with *a* = −1 can be represented as:

(21) $$E_{m}\left(\varphi ,a=-1\right)=\left(\begin{array}{l} \cos\left(m\varphi \right)+1\\ \sin\left(m\varphi \right) \end{array}\right)=2\cos\left(\frac{m\varphi }{2}\right)\left(\begin{array}{l} \cos\left(\frac{m\varphi }{2}\right)\\ \sin\left(\frac{m\varphi }{2}\right) \end{array}\right).$$

It can be seen from (21) that the initial field (1) for *a* = −1 and odd *m* is a cylindrical vector field of fractional order (*m*/2). It was shown in [10] that such a light field has local regions with a vortex energy flow and with a circular polarization in the focus.

The formation of local regions with a circular and an elliptical polarization in the focus of the field (1) follows from the expressions for the electric vector projections in the focus (2). It can be seen from (2) that at *a* = 0 (there is no field with linear polarization), the product of the electric vector transverse projections will be real Im(Ex∗Ey)=0 since both projections will have the same factor ${\left(-i\right)}^{m-1}$. If a ≠ 0, then both expressions for the transverse projections of the electric vector in (2) consist of two terms with factors ${\left(-i\right)}^{m-1}$ and *i*. Therefore, the product $E_{x}^{*}E_{y}$ will have a factor ${\left(-i\right)}^{m}$ that is imaginary for the odd *m*. Therefore, the longitudinal projection of the SAM or the third Stokes component will be different from zero Im(Ex∗Ey)≠0. This means that there are local regions in the focus in which the polarization is elliptical or circular.

## 3. Numerical Simulations Results and Discussion

**Figure 1:**
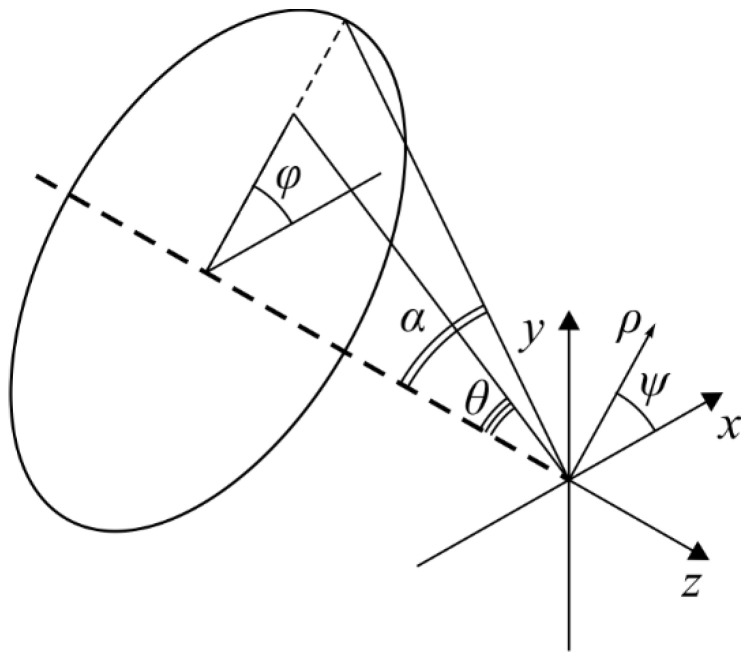
The geometric interpretation of the problem.

In this section, we present the simulation results for the intensity distribution, the projections of the Stokes vectors (or the SAM longitudinal projection), as well as the Poynting vector projections in the focus of the initial light field (1). The calculation was carried out using the general formulas of Richards-Wolf [12], which describe the light in the focus area:

(22) $$\begin{array}{l} U\left(\rho ,\psi ,z\right)=-\frac{if}{\lambda }\int_{0}^{\alpha }\int_{0}^{2\pi }B\left(\theta ,\varphi \right)T\left(\theta \right)P\left(\theta ,\varphi \right)\times \\ \times \exp\left\{ik\left[\rho \sin\theta \cos\left(\varphi -\psi \right)+z\cos\theta \right]\right\}\sin\theta \;d\theta \;d\varphi , \end{array}$$

where **U**(ρ, ψ, *z*) is an electric or magnetic field, *B*(θ, φ) is an electric or a magnetic field at the input of a wide-aperture optical system dependent on the exit pupil coordinates (θ is a polar angle, φ is an azimuth angle), *T*(θ) is a lens apodization function, *f* is a focal length, *k*  =  2π/λ is a wave number, λ is a wavelength, α is the maximum polar angle defined by the lens numerical aperture (NA  =  sinα) and **P**(θ, φ) is a polarization matrix. Integral (22) allows us to calculate the distribution of the electromagnetic field components in the exit pupil coordinates (Figure 1).

The polarization matrix **P**(θ, φ) for the electric and magnetic fields has the form [18,19]:

(23) $$P(\theta ,\phi )=\left[\begin{array}{ccc} \sin^{2}\phi +\cos^{2}\phi \cos\theta & \sin\phi \cos\phi \left(\cos\theta -1\right) & \cos\phi \sin\theta \\ \sin\phi \cos\phi \left(\cos\theta -1\right) & \cos^{2}\phi +\sin^{2}\phi \cos\theta & \sin\phi \sin\theta \\ -\sin\theta \cos\phi & -\sin\theta \sin\phi & \cos\theta \end{array}\right]\times \left[\begin{array}{c} a\left(\theta ,\phi \right)\\ b\left(\theta ,\phi \right)\\ c\left(\theta ,\phi \right) \end{array}\right]$$

where *a*(θ,ϕ), *b*(θ,ϕ) and *c*(θ,ϕ) are polarization functions for the *x*-, *y*- and *z*-component of an incident field. For example, for the linearly polarized along the *x*-axis light, the components will be equal to *a* = 1, *b* = 0 and *c* = 0. For all the examples considered in this section, the longitudinal component of the focused field was proposed to be zero: *c* = 0 (the initial plane), then:

(24) $$P\left(\theta ,\varphi \right)=\left[\begin{array}{c} 1+\cos^{2}\varphi \left(\cos\theta -1\right)\\ \sin\varphi \cos\varphi \left(\cos\theta -1\right)\\ -\sin\theta \cos\varphi \end{array}\right]a\left(\theta ,\varphi \right)+\left[\begin{array}{l} \sin\varphi \cos\varphi \left(\cos\theta -1\right)\\ 1+\sin^{2}\varphi \left(\cos\theta -1\right)\\ -\sin\theta \sin\varphi \end{array}\right]b\left(\theta ,\varphi \right),$$

For the initial field (1), the polarization functions will have the form:

(25) $$E(\theta ,\varphi )=\left(\begin{array}{l} a\left(\theta ,\varphi \right)\\ b\left(\theta ,\varphi \right) \end{array}\right)=\left(\begin{array}{l} \cos\left(m\varphi \right)-a\\ \sin\left(m\varphi \right) \end{array}\right)$$

for the electric field and

(26) $$H\left(\theta ,\varphi \right)=\left(\begin{array}{l} a\left(\theta ,\varphi \right)\\ b\left(\theta ,\varphi \right) \end{array}\right)=\left(\begin{array}{l} -\sin\left(m\varphi \right)\\ \cos\left(m\varphi \right)-a \end{array}\right)$$

for the magnetic field.

### 3.1. The Distribution of Linear Polarization Vectors in the Initial Plane

**Figure 2:**
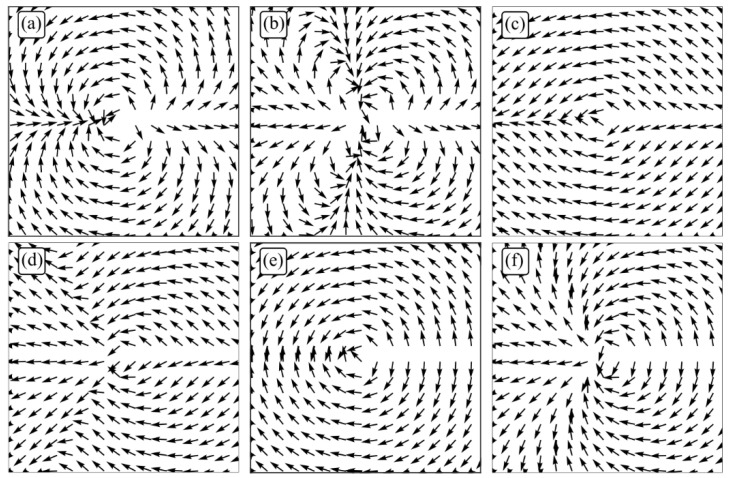
The distributions of linear polarization vectors in the cross section of the field (1) for *m* = 2 (**a**,**c**,**e**) and *m* = 3 (**b**,**d**,**f**), and *a* = 1/2 (**a**,**b**), *a* = 3/2 (**c**,**d**), *a* = 1 (**e**,**f**).

The distribution of linear vectors over the beam cross-section in the initial plane depends on the number *m* and the parameter *a*. Figure 2 shows the distributions of linear polarization vectors in the cross-section of the field (1) for *m* = 2 (a, c, e) and *m* = 3 (b, d, f), and *a* = 1/2 (a, b), *a* = 3/2 (c, d), *a* = 1 (e, f).

It was shown in [13] that the polarization singularity index (the number of linear polarization vector rotations by 2π while going around the closed contour around the optical axis—around the singularity point) at *a* < 1 is equal to *m*. Thus, the polarization singularity index is equal to 2 (a) and 3 (b) in Figure 2. The polarization singularity index is equal to *m*/2 for *a* = 1 since there are only singularity lines (Figure 2e,f) in the field (1), and the linear polarization vectors rotate from line to line by an angle *π*. Therefore, the index is equal to 1 (e) and 3/2 (f) in Figure 2. For *a* > 1, the polarization singularity index of the field (1) is equal to zero (Figure 2c,d).

### 3.2. The Intensity Distribution in the Focal Plane

**Figure 3:**
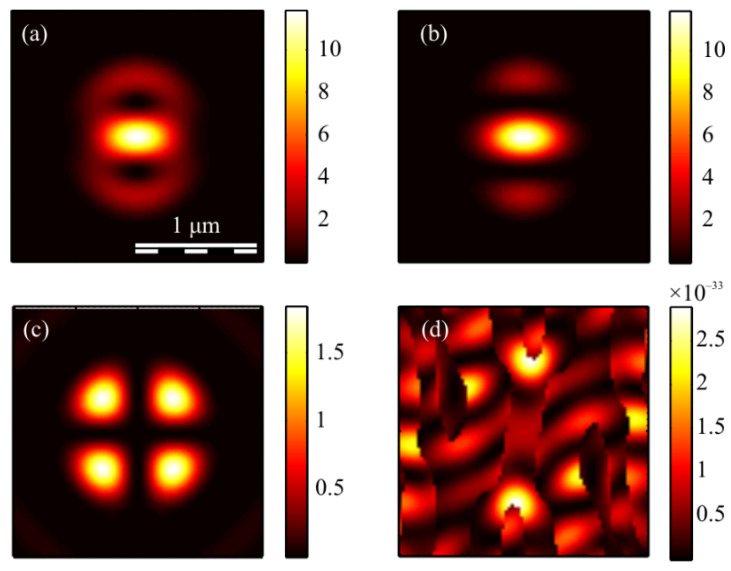
The intensity *I* (**a**) and its components *I_x_* (**b**), *I_y_* (**c**) *I_z_* (**d**) of the focused vector field (1) of the second (*m* = 2) order with *a* = 1.

**Figure 4:**
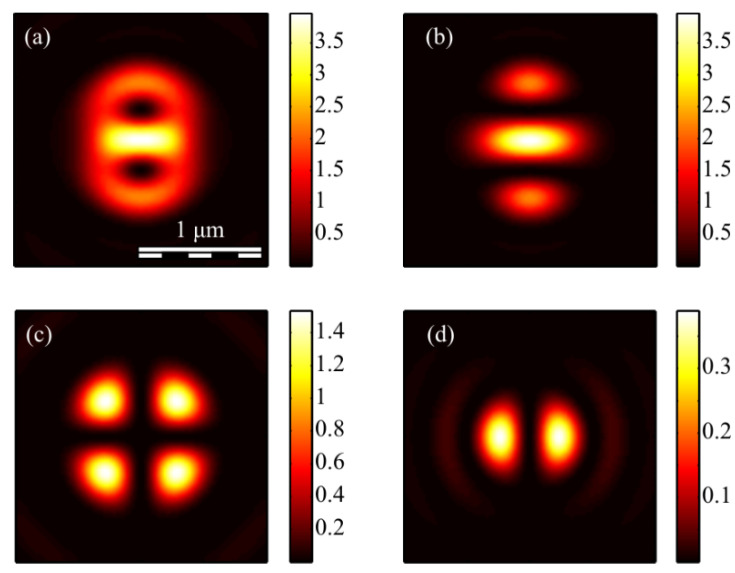
The intensity *I* (**a**) and its components *I_x_* (**b**), *I_y_* (**c**) *I_z_* (**d**) of the focused vector field (1) of the second (*m* = 2) order with *a* = 1/2.

**Figure 5:**
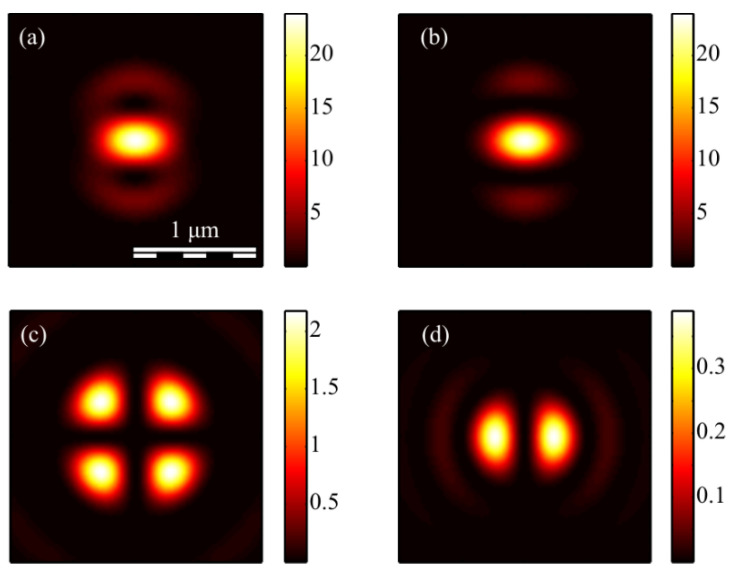
The intensity *I* (**a**) and its components *I_x_* (**b**), *I_y_* (**c**) *I_z_* (**d**) of the focused vector field (1) of the second (*m* = 2) order with *a* = 3/2.

**Figure 6:**
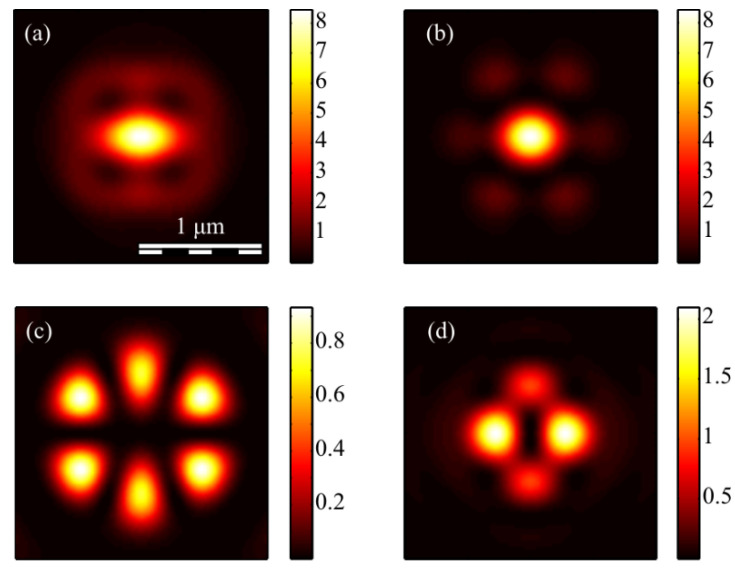
The intensity *I* (**a**) and its components *I_x_* (**b**), *I_y_* (**c**) *I_z_* (**d**) of the focused vector field (1) of the second (*m* = 3) order with *a* = 1.

**Figure 7:**
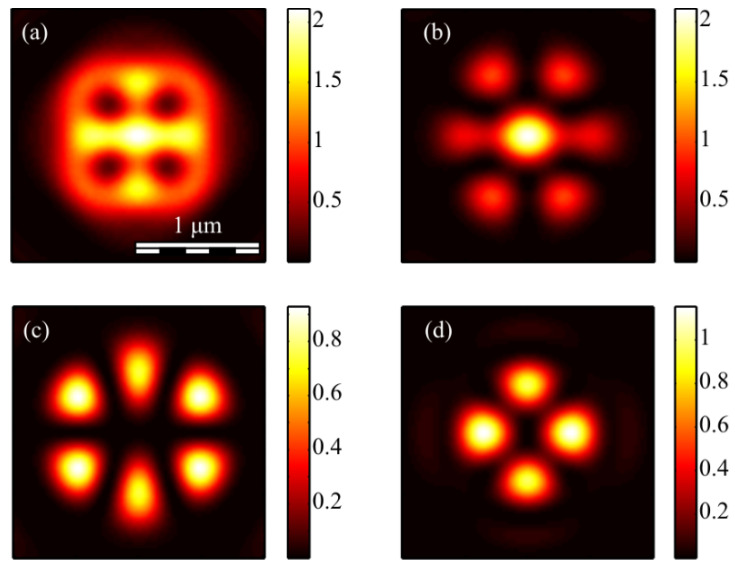
The intensity *I* (**a**) and its components *I_x_* (**b**), *I_y_* (**c**) *I_z_* (**d**) of the focused vector field (1) of the second (*m* = 3) order with *a* = 1/2.

**Figure 8:**
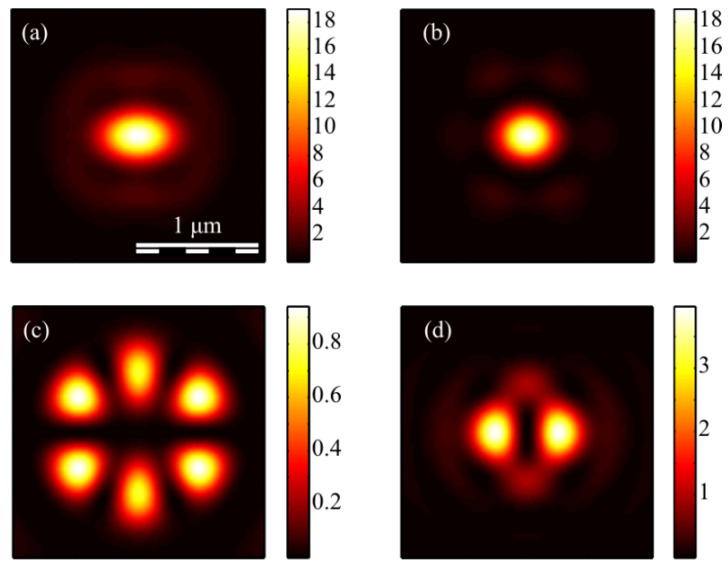
The intensity *I* (**a**) and its components *I_x_* (**b**), *I_y_* (**c**) *I_z_* (**d**) of the focused vector field (1) of the second (*m* = 3) order with *a* = 3/2.

The calculation of the intensity in the focus of the field (1) was carried out using the Richards–Wolf formulas (22)–(26) for the wavelength of 633 nm and the numerical aperture *NA* = 0.95. The intensity distribution and its components were calculated for the vector beam (1) of the second (*m* = 2) (Figure 3, Figure 4 and Figure 5) and third (*m* = 3) (Figure 6, Figure 7 and Figure 8) orders. The beam parameter *a* (1) was chosen to be 1 (Figure 3 and Figure 6), 1/2 (Figure 4 and Figure 7) and 3/2 (Figure 5 and Figure 8).

Figure 3a, Figure 4a and Figure 5a show that for any *a* > 0, an elliptical focal spot elongated along the *x-*axis is formed near the optical axis in the center of the focus. The intensity maximum on the optical axis follows from formulas (6)–(9). The intensity distributions in Figure 3a, Figure 4a and Figure 5a differ only by the size of the central and two side lobes. For *a* > 1, the side lobes are small, and almost all the intensity is in the central elliptical spot. For *a* = 1, the energy of the side lobes increases. For *a* < 1, the energy of the two side lobes, whose intensity maxima lie on the vertical *y*-axis, is comparable to the intensity of the central focal spot. A feature of the intensity distribution in Figure 3, Figure 4 and Figure 5 is that the longitudinal component is absent for the case of *a* = 1. This unique case takes place only when *m* = 2 and *a* = 1 and is described by the formula (8). Figure 3d, Figure 4d and Figure 5d confirm the formula (7), according to which the longitudinal intensity *I_z_* at *a* ≠ 1 has two local intensity maxima on the horizontal axis at φ = 0 and φ = π. There are four local maxima for any *a* in Figure 3c, Figure 4c and Figure 5c. This is consistent with the formula (6) since *I_y_* should have 2*m* such maxima. Figure 6, Figure 7 and Figure 8 show intensity distributions similar to those shown in Figure 3, Figure 4 and Figure 5 but for the odd *m* = 3.

Figure 6a, Figure 7a and Figure 8a show a focal elliptical spot with side lobes in the center of the intensity pattern near the optical axis. The number of lobes, according to the formula (9), is 2(*m* − 1) = 4. These lobes are clearly visible in Figure 7a, where the parameter *a* < 1 and almost invisible in Figure 8a, where *a* > 1. Figure 6d, Figure 7d and Figure 8d confirm the formula (7), according to which the longitudinal intensity *I_z_* has four local intensity maxima for any *a*. Two maxima located on the horizontal *x*-axis are larger in magnitude than two maxima on the vertical *y*-axis. There are six local maxima for any *a* in Figure 6c, Figure 7c and Figure 8c. This is consistent with the formula (6) since *I_y_* should have 2*m* such maxima.

### 3.3. The Distribution of the Stokes Vector Projections in the Focal Plane

**Figure 9:**
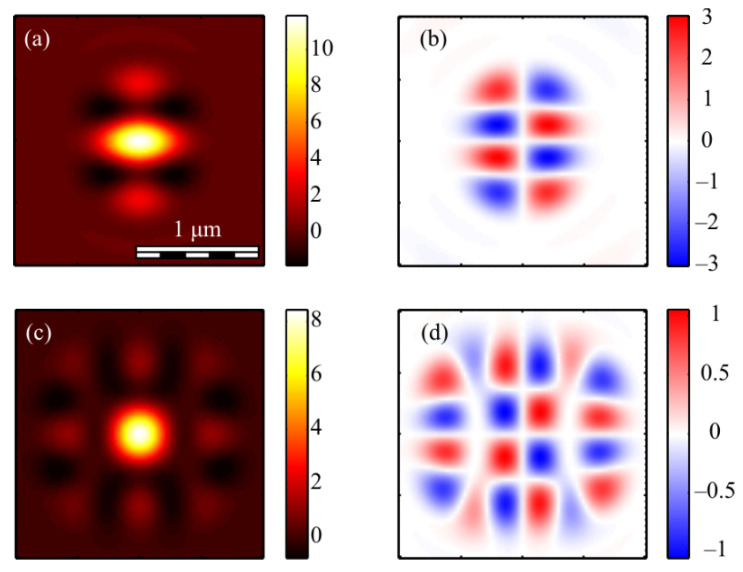
The Stokes vector components *s*_1_ (**a**,**c**) and *s*_2_ (**b**,**d**) of the focused vector field (1) with *m* = 2 (**a**,**b**) and *m* = 4 (**c**,**d**) for *a* = 1.

It can be seen from (19) that the third projection of the Stokes vector is equal to zero for even *m*. This means that the polarization is linear at each point of the field in the focal plane. Figure 9 shows projections of the non-normalized Stokes vector *s*_1_ and *s*_2_ (*s*_3_ = 0) for even numbers *m* = 2 (a, b) and *m* = 4 (c, d).

It can be seen from Figure 9a that the distribution pattern *s*_1_ almost coincides with *I_x_* (Figure 4b). This is because *s*_1_ = *I_x_* − *I_y_* and *I_x_* > *I_y_*.

**Figure 10:**
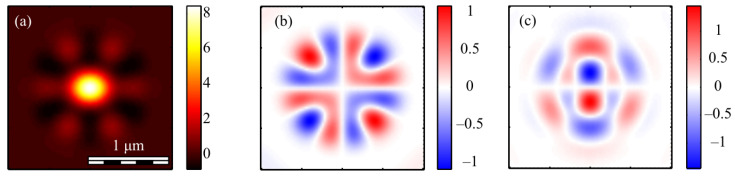
The Stokes vector components *s*_1_ (**a**), *s*_2_ (**b**), and *s*_3_ (**c**) of the focused vector field (1) with *m* = 3 and *a* = 1.

The third projection of the Stokes vector is non-zero in the focus only for an odd number *m*. Figure 10 shows three projections of the non-normalized Stokes vector in the focus of field (1) with *m* = 3 and *a* = 1.

Figure 10a shows that the distribution pattern of *s*_1_ almost coincides with *I_x_* (Figure 7c). This is because *s*_1_ = *I_x_* − *I_y_* and *I_x_* > *I_y_*. It can be seen from Figure 10c that the third projection of the Stokes vector *s*_3_ changes sign 2*m* = 6 times on circles with certain radii and with center on the optical axis. This is consistent with the formula (19), which includes sin(*m*φ). This function changes the sign 2*m* times per turn. The second Stokes projection changes sign when going around a closed trajectory around the optical axis 4*m* times: 8 (Figure 9b), 16 (Figure 9d) and 12 (Figure 10b). This is consistent with the formula (20), in which the term with the maximum argument has the form sin(2*m*φ).

**Figure 11:**
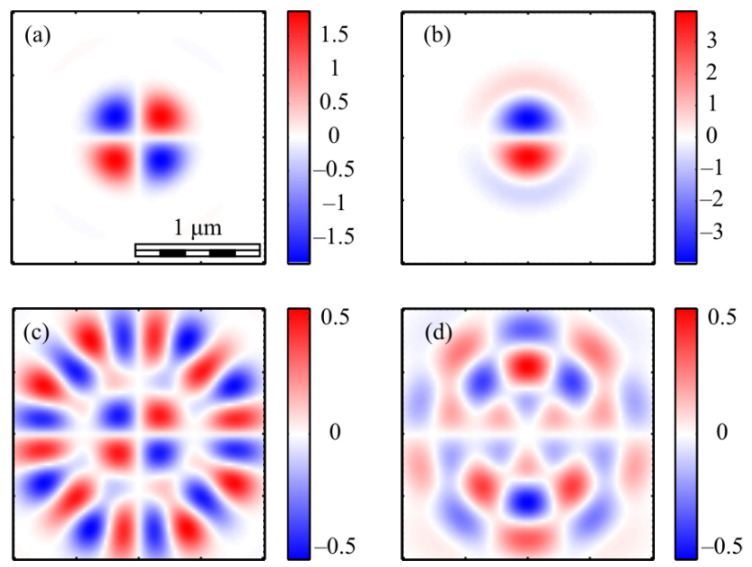
The Stokes vector components s_2_ (**a**,**c**) and s_3_ (**b**,**d**) of the focused vector field (1) with *m* = 1 (**a**,**b**) and *m* = 5 (**c**,**d**).

For comparison, Figure 11 shows the second *s*_2_ (a, c) and the third *s*_3_ (b, d) components of the Stokes vector of the focused vector field (1) with other odd numbers *m*: 1 (a, b) and 5 (c, d).

It can be seen from Figure 11 that the distribution *s*_2_ changes sign 4*m* times when going around the optical axis: 4 (Figure 11a) and 20 (Figure 11c). This is consistent with the formula (20). Additionally, the distribution *s*_3_ changes sign 2*m* times: 2 (Figure 11b) and 10 (Figure 11d). This is consistent with the formula (19).

**Figure 12:**
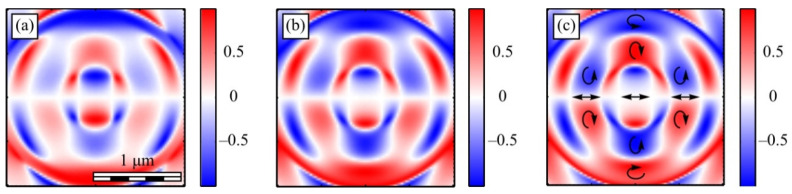
The third component of the normalized Stokes vector (16) *S_3_* of the focused vector field (1) with *m* = 3 and different parameters *a*: 1/2 (**a**), 1 (**b**) and 3/2 (**c**). Arrows indicate the circular, elliptical or linear polarization.

For the sake of completeness, we show the distributions of the third component of the normalized Stokes vector (Figure 12).

Figure 12 shows that the magnitude and the size of the regions with an elliptical and a circular polarization, where the component *S_3_* is close to +1 (red color) or −1 (blue color), decreases with the decreasing parameter *a*. The comparison of Figure 10c and Figure 12 show that the structures of the normalized *S_3_* and the non-normalized *s*_3_ qualitatively agree.

In this subsection, it is shown by numerical examples that local regions (size is about 200–250 nm) with an elliptical or a circular polarization are formed in the focus of the vector field (1) (wavelength is 633 nm, *NA* = 0.95). The number of such regions is related to the field number *m*. The number of such regions is 2*m* on some circles centered on the optical axis. It should be noted that the regions with the local elliptical polarization appear in the focus only for an odd number *m* and for a non-zero parameter *a*. If the parameter *a* = 0, then the field (1) reduces to the well-known cylindrical vector field of order *m*, which has for any *m* only a local linear polarization in the focus, and regions with an elliptical polarization are absent.

### 3.4. The Distribution of the Poynting Vector Projections in the Focal Plane

This section presents the calculation of the energy flux vector (the Poynting vector) (10) in the tight focus of the field (1) carried out by the Richards–Wolf formulas (22)–(26).

**Figure 13:**
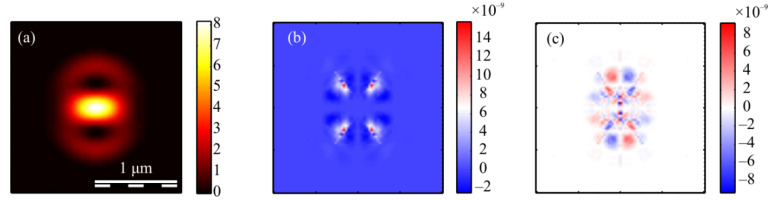
The Poynting vector components in the focus for *m* = 2 and *a* = 1: *P_z_* (**a**), *P_x_* (**b**), P_y_ (**c**).

**Figure 14:**
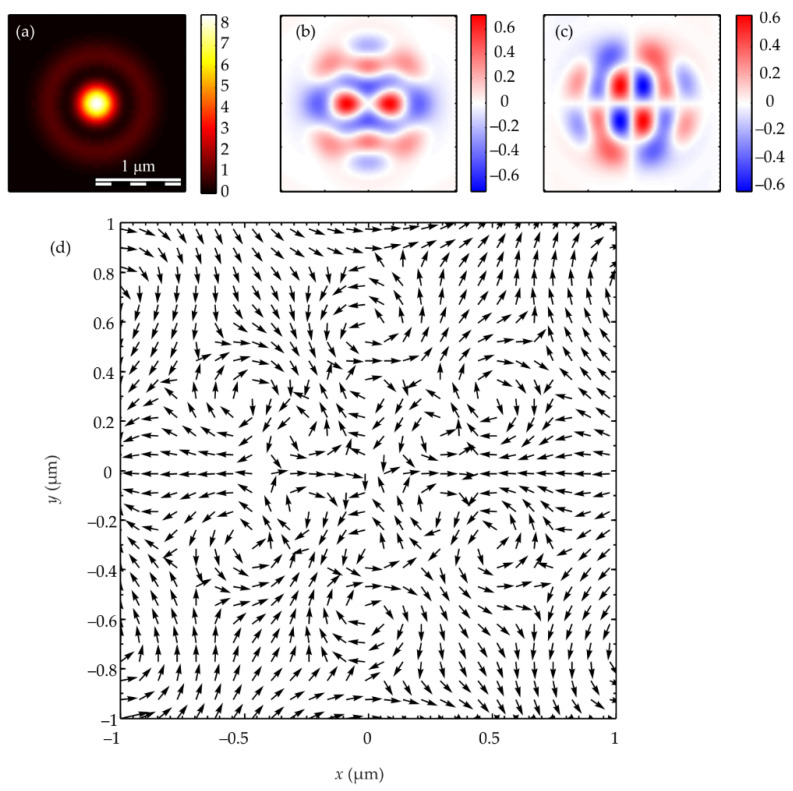
The Poynting vector components in the focus for *m* = 3 and *a* = 1: *P_z_* (**a**), *P_x_* (**b**), P_y_ (**c**). The arrows show the direction of the transverse Poynting vector in the focus (**d**).

Figure 13 shows the projections of the Poynting vector in the focus of the field (1) with the even number *m =* 2 and *a* = 1. According to the obtained even number *m* formulas (11)–(13), the transverse projections of the energy flux vector *P_x_* and *P_y_* are equal to zero (Figure 13b,c), and the longitudinal component *P_z_* does not have a radial symmetry (Figure 13a). It can be seen from Figure 13a and the formula (13) that the Poynting vector longitudinal component has a local maximum on the optical axis, and two local maxima (side lobes) are located on the vertical axis at φ = π/2 and φ = 3π/2 since the function *P_z_* (13) for *m* = 2 depends on the angle as cos(2φ). The calculation parameters in Figure 13 and Figure 14 are the same as in all previous Figures. Figure 13a also shows that the longitudinal component of the Poynting vector outwardly coincides with the intensity distribution in Figure 3a (*m* = 2). This is explained by the fact that the expression for the intensity (9) at *m* = 2, as well as (13), depends on the angle as cos(2φ).

Figure 14 shows the Poynting vector projections for the odd number *m* = 3 and *a* = 1 in the focus. It can be seen in Figure 14a that the longitudinal component *P_z_* is radially symmetric and has a maximum value on the optical axis. This is consistent with the Equation (13). It can be seen from Figure 14b,c that the transverse energy flow rotates in eight local subwavelength regions: counterclockwise in four regions and clockwise in the other four regions. Both transverse projections of the energy flux *P_x_* and *P_y_* change the sign eight times when going around the optical axis along a circle of some radius. This is consistent with formulas (11) and (12) since the dependence on the angle in these formulas is determined by the function cos((*m* + 1)φ) at *m* = 3. However, the number of local areas with a transverse vortex flow will be 2*m*. This follows from Equation (15), in which there are factors cos(*m*φ) and sin(*m*φ). The number of local regions with a vortex energy flow must be equal to the number of regions with a circular polarization, i.e., 2*m*. This follows from the effect of the spin-orbital interaction. There are six such regions in Figure 14d, and they lie along a circle of some radius. Their size is about 200 nm. Integrating in (15) the angular *P*_φ_ and the radial *P_r_* projections of the Poynting vector over the angle φ, we obtain that the total transverse energy flux is equal to zero in the focus.

Comparing Figure 10c and Figure 14d shows that there are six regions lying on a circle of a certain radius centered on the optical axis, in which the polarization is circular, and there are also six regions in which the energy flow rotates around the ring.

**Figure 15:**
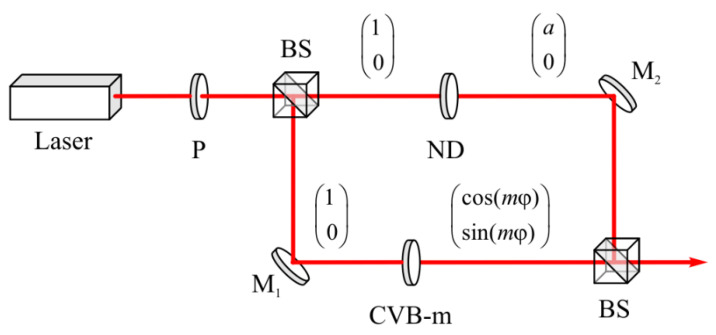
Optical setup for the generation of a beam with a non-uniform polarization (1). P is a polarizer; BS is a beam splitter; ND is a neutral density filter; M1 and M2 are mirrors; CVB m is a vector wave plate.

Figure 15 shows the optical scheme for the beam (1) generation. The beam of light from the laser passes through the polarizer P and becomes linearly polarized along the horizontal axis. The initial light is divided into two identical beams after dividing cube BS. In one arm of the Mach–Zender interferometer, the beam passes through the *m*-th order q-plate, and the *m*-th order cylindrical vector beam (CVB-m) is formed. In the other arm of the interferometer, the amplitude of the beam with linear polarization is changed so that it is equal to *a*. After the second dividing cube BS, both beams are combined into one beam, the amplitude of which is proportional to the beam (1).

## 4. Conclusions

In this paper, using the Richards–Wolf approach, all six projections of an electric and a magnetic field vectors in the tight focus of a superposition of a cylindrical vector field with order *m* and a uniform field with linear polarization were theoretically and numerically calculated. Energy fluxes (projections of the Poynting vector), intensity distributions and Stokes components were calculated in the focus plane. The study shows that local regions of about 200–250 nm in size (the wavelength is 633 nm, *NA* = 0.95) with an elliptical or a circular polarization are formed in the focal plane of such an incident field. The number of such areas is related to the field number *m*. The number of such regions on some circles with a center on the optical axis is 2*m* in the focus. It should be noted that regions with the local elliptical polarization only appear in the focus for an odd number *m* and for a non-zero parameter *a*. If the parameter *a* of the initial field is zero, then the field reduces to the well-known cylindrical vector field of order *m*, which, for any *m*, only has a local linear polarization in the focus. Additionally, regions with an elliptical polarization are absent in this case. In addition to the presence of a local SAM for the case of an odd number *m*, there are local subwavelength regions in the focus where the transverse energy flux propagates along a closed contour. The number of such regions lying on a circle of some radius is 2*m*. Moreover, the energy flow in neighboring areas rotates in different directions (clockwise and counterclockwise). The total transverse energy flow is zero. These beams can be used to create a micromachine in which two microparticles in the form of gears are captured in the focus of the beam into neighboring local areas in which the energy flow rotates in different directions, and therefore, these gears will also rotate in different directions.

## References

[1] Zhan Q., Leger J.R. (2002). Focus shaping using cylindrical vector beams. Opt. Express.

[2] Zhan Q. (2009). Cylindrical vector beams: From mathematical concepts to applications. Adv. Opt. Photonics.

[3] Machavariani G., Lumer Y., Moshe I., Meir A., Jackel S. (2007). Efficient extracavity generation of radially and azimuthally polarized beams. Opt. Lett..

[4] Liu Z., Liu Y., Ke Y., Liu Y., Shu W., Luo H. (2017). Generation of arbitrary vector vortex beams on hybrid-order Poincare sphere. Photonics Res..

[5] Liu J., Chen X., He Y., Lu L., Ye H., Chai G., Chen S., Fan D. (2020). Generation of arbitrary cylindrical vector vortex beams with cross-polarized modulation. Results Phys..

[6] Yan S., Yao B. (2007). Radiation forces of a highly focused radially polarized beam on spherical particles. Phys. Rev. A.

[7] Chen R., Agarwal K., Sheppard C.J., Chen X. (2013). Imaging using cylindrical vector beams in a high-numerical-aperture microscopy system. Opt. Lett..

[8] Fickler R., Lapkiewicz R., Ramelow S., Zeilinger A. (2014). Quantum entanglement of complex photon polarization patterns in vector beams. Phys. Rev. A.

[9] Hollezek A., Aiello A., Gabriel C., Morquargt C., Leuchs G. (2011). Classical and quantum properties of cylindrically polarized states of light. Opt. Express.

[10] Stafeev S.S., Nalimov A.G., Zaitsev V.D., Kotlyar V.V. (2021). Tight focusing cylindrical vector beams with fractional order. J. Opt. Soc. Am. B.

[11] Kotlyar V.V., Nalimov A.G., Stafeev S.S. (2019). Exploiting the circular polarization of light to obtain a spiral energy flow at the subwavelength focus. J. Opt. Soc. Am. B.

[12] Richards B., Wolf E. (1959). Electromagnetic Diffraction in Optical Systems. II. Structure of the Image Field in an Aplanatic System. Proc. R. Soc. A Math. Phys. Eng. Sci..

[13] Kotlyar V.V., Kovalev A.A., Zaitsev V.D. (2022). Topological Charge of Light Fields with a Polarization Singularity. Photonics.

[14] Freund I. (2002). Polarization singularity indices in Gaussian laser beams. Opt. Commun..

[15] Kotlyar V.V., Stafeev S.S., Kovalev A.A. (2019). Sharp focusing of a light field with polarization and phase singularities of an arbitrary order. Comput. Opt..

[16] Bliokh K.Y., Ostrovskaya E.A., Alonso M.A., Rodriguez-Herrera O.G., Lara D., Dainty C. (2011). Spin-to-orbital angular momentum conversion in focusing, scattering, and imaging systems. Opt. Express.

[17] Born M., Wolf E. (1973). Principles of Optics.

[18] Pereira S.F., Van de Nes A.S. (2004). Superresolution by means of polarisation, phase and amplitude pupil masks. Opt. Commun..

[19] Khonina S.N., Volotovsky S.G. (2010). Control by contribution of components of vector electric field in focus of a high-aperture lens by means of binary phase structures. Comput. Opt..

